# The role of oceanographic conditions and colony size in shaping the spatial structure of *Pyrosoma atlanticum* in the NW Mediterranean Sea

**DOI:** 10.1093/plankt/fbac056

**Published:** 2022-10-12

**Authors:** Marina Pastor-Prieto, Ana SabatÉs, Vanesa Raya, Antonio Canepa, TomÁs I Parraguez, Josep-Maria Gili

**Affiliations:** Institut de Ciències del Mar (ICM-CSIC), Passeig Marítim de la Barceloneta, 37-49, Barcelona 08003, Spain; Institut de Ciències del Mar (ICM-CSIC), Passeig Marítim de la Barceloneta, 37-49, Barcelona 08003, Spain; Institut de Ciències del Mar (ICM-CSIC), Passeig Marítim de la Barceloneta, 37-49, Barcelona 08003, Spain; Departamento de Ingeniería Informática, Escuela Politécnica Superior, Universidad de Burgos, Avda. Cantabria, Burgos 09006, Spain; Institut de Ciències del Mar (ICM-CSIC), Passeig Marítim de la Barceloneta, 37-49, Barcelona 08003, Spain; Institut de Ciències del Mar (ICM-CSIC), Passeig Marítim de la Barceloneta, 37-49, Barcelona 08003, Spain

**Keywords:** diel vertical migration, mesoscale distribution, ontogenetic stage, shelf-slope front, gelatinous zooplankton

## Abstract

This study investigates the role of winter oceanographic conditions on the horizontal and vertical spatial structure of *Pyrosoma atlanticum* at different ontogenetic stages. Data were obtained on two oceanographic cruises (February 2017 and 2018) in the NW Mediterranean. Small colonies were exceptionally abundant in 2017, linked to an earlier development of spring conditions and the subsequent seasonal phytoplankton bloom. The mesoscale distribution of *P. atlanticum* differed depending on the colony size. Large colonies (≥7 mm) were found on the slope all along the density front, whereas small (<4 mm) and medium colonies (4–6.9 mm) extended their distribution over the shelf because of instabilities of the front, and were mostly absent in the cold, low-salinity coastal waters. The analysis of their vertical distribution showed that at night colonies of all sizes remained close to the surface, where chlorophyll-*a* levels were high, whereas during the day they migrated to deeper layers, reaching greater depths as the colony size increased. The migratory behaviour started when colonies were 4–6.9 mm long. The relative importance of the species in the downward carbon transport is discussed. Our results highlight the need to further study the ecology of this efficient filter feeder in the Mediterranean.

## INTRODUCTION

Pyrosomes (Greek for “fire bodies” because of their bioluminescence) are colonial pelagic tunicates made up of tens to thousands of zooids encased in a common gelatinous tunic (Godeaux *et al*., 1998; Madin and Deibel, 1998) ranging from <1 cm to a maximum recorded length of 20 m, depending on the species (Van Soest, 1981). These colonies are holoplanktonic grazers, feeding mainly on phytoplankton of a wide range of types and sizes (Drits *et al*., 1992; Perissinotto *et al*., 2007; Décima *et al*., 2019). As with other pelagic tunicates, pyrosomes have high clearance rates that result in a substantial energy transfer to deep waters (Henschke *et al*., 2019) through their large production of faecal pellets (Drits *et al*., 1992) and carcass depositions (Lebrato and Jones, 2009). Pyrosomes are preyed by sea lions, fish, turtles and seabirds in the water column (Harbison, 1998; Childerhouse *et al*., 2001; Hedd and Gales, 2001), and by arthropods, cnidarians, fish and sharks on the seafloor (Carrassón and Cartes, 2002; Lebrato and Jones, 2009; Archer *et al*., 2018; Brodeur *et al*., 2021). These biological traits and trophic interactions give pyrosomes an important role in the marine trophic web and carbon transport (Lebrato and Jones, 2009; Henschke *et al*., 2019).

*Pyrosoma atlanticum* is the most widespread and common species. Historically, it has been found in open waters of all oceans between 50°N and 50°S (Van Soest, 1981), but it has recently been reported further north in the Pacific linked to a large marine heat wave (Brodeur *et al*., 2018; Miller *et al*., 2019). As found in other gelatinous zooplankton, hydrodynamic structures such as currents, gyres and fronts drive their transport and concentration, ultimately shaping their areas of distribution and abundance (Graham *et al*., 2001; Guerrero *et al*., 2016; Bellido *et al*., 2020). Physical and biological gradients in the water column (e.g. thermoclines and subsurface chlorophyll maxima) determine the vertical distribution of these organisms, limiting their movement or leading to an increase in abundance that results in a patchy distribution (Gibbons *et al*., 1999; McManus *et al*., 2003; Nogueira Júnior *et al*., 2015). *P. atlanticum* is known as a strong migrator that can reach up to 2500 m depth during the day and migrates towards the surface at night (Roe *et al*., 1987; Angel, 1989). Its short generation time and rapid growth, together with its high filtration rates, allow an exponential population increase of *P. atlanticum* under favourable environmental conditions (Alldredge and Madin, 1982), in some cases leading to large swarms (Angel, 1989; Drits *et al*., 1992). Unprecedented high densities of large colonies of *P. atlanticum* were reported in the northeast Pacific associated with a warm water mass and a strong El Niño (Brodeur *et al*., 2018; Sutherland *et al*., 2018). Despite observations of aggregations in the Tasman Sea (Henschke *et al*., 2019), southeast Atlantic (Drits *et al*., 1992), Gulf of Mexico (Archer *et al*., 2018) and Mediterranean Sea (Braconnot and Goy, 1981), the environmental drivers of these blooms remain unclear.

The Mediterranean Sea has a marked seasonal cycle, with the alternation of stratified (summer) and mixed (winter) periods that confers strong seasonality to primary production (Estrada *et al*., 1985). Recurrent late winter–early spring blooms are only observed regularly in the northwestern region and intermittently in a few other areas (D’Ortenzio and Ribera d’Alcalà, 2009). The seasonal bloom in the NW Mediterranean is triggered by deep water formation episodes that take place in the Gulf of Lions, driven by evaporation caused by strong, cold and dry northerly winds (MEDOC group, 1970; Schott *et al*., 1996). Interannual variability in primary production is highly dependent on the extent, intensity and duration of the deep water formation episodes, which increase in colder and drier years (Marty and Chiavérini, 2010; Herrmann *et al*., 2013). The dynamics of the NW Mediterranean is characterized by the presence of a permanent shelf-slope density front along the slope separating open sea high-salinity waters from coastal low-salinity waters (Font *et al*., 1988). A geostrophic current (the Northern Current) associated with the front flows from NE to SW, roughly parallel to the coast (Millot, 1999). The front and the associated current are subject to high mesoscale variability that causes oscillations, meandering and eddy generation (Sabatés *et al*., 2004; Rubio *et al*., 2005), playing a key role in the distribution and abundance of planktonic organisms (e.g. Sabatés *et al*., 2004; Guerrero *et al*., 2016).

Previous studies carried out on gelatinous zooplankton in the NW Mediterranean have shown high spatial variability closely linked to oceanographic dynamics and water mass structure (Guerrero *et al*., 2016; Sabatés *et al*., 2018). At a temporal scale, high interannual variability has been reported in abundance and species composition (Licandro and Ibanez, 2000; Guerrero *et al*., 2018b; Feuilloley *et al*., 2021), but there is no clear consensus on long-term trends (Molinero *et al*., 2005; García-Comas *et al*., 2011; Licandro *et al*., 2012; Guerrero *et al*., 2018a). Studies on *P. atlanticum* in the Mediterranean are scarce. Although some sporadic records of the species have been reported in the eastern basin (Galil and Goren, 1994), most of the studies have been conducted in the western basin. These studies have addressed the vertical distribution (Palma, 1985; Andersen and Sardou, 1994; Sardou *et al*., 1996), seasonality and population dynamics (Franqueville, 1971; Braconnot, 1974) of *P. atlanticum* mainly from fixed stations or single transects in which sampling was performed in spring. In this study, we addressed the spatial structure of *P. atlanticum* in the NW Mediterranean during winter oceanographic conditions. The specific objectives were (i) to analyse how the mesoscale water dynamics shape the horizontal distribution of *P. atlanticum* at different ontogenetic stages; and (ii) to determine diel and ontogenetic changes in vertical distributions of *P. atlanticum* in relation to the structure of the water column.

## METHOD

### Field sampling

**Fig. 1:**
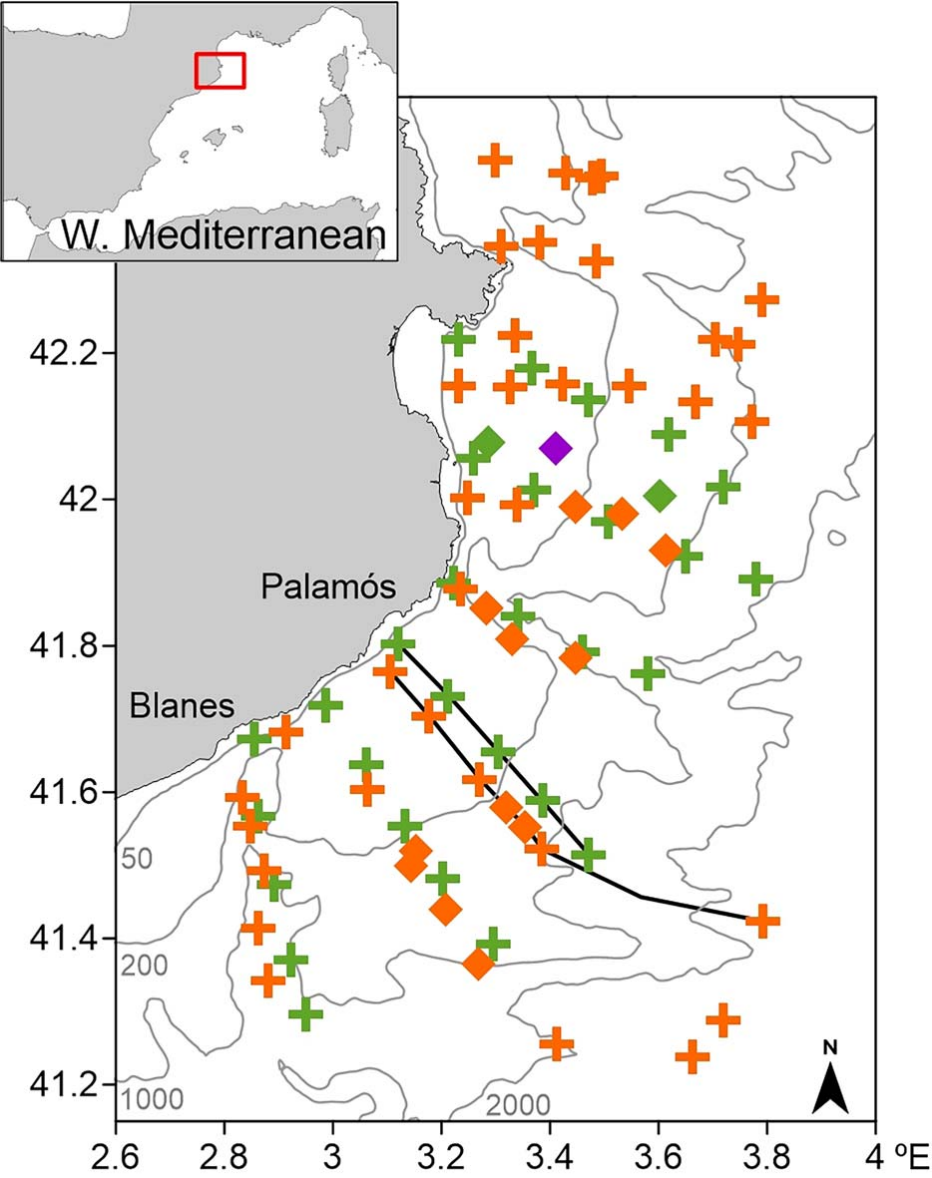
Study area in the NW Mediterranean. Sampling stations during 2017 (orange) and 2018 (green) surveys. Lines indicate the location of the vertical sections in Fig. 3. Diamonds represent depth-stratified samplings, the purple one corresponds to a fixed station sampled for 48 h in the 2017 survey. Thin grey lines show isobaths (50, 200, 1000 and 2000 m).

Two oceanographic cruises were conducted in the NW Mediterranean (41.3–42.5°N and 2.8–3.8°E) during two consecutive winters (18 February–20 March 2017 and 18–28 February 2018) on board the R/V García del Cid. Sampling stations were placed along transects perpendicular to the coast located at 7–14 km apart and covering the shelf and slope regions (Fig. 1). At each station, vertical profiles of basic hydrographic variables (salinity, temperature and fluorescence) were obtained by means of a conductivity-temperature-depth (CTD) profiler equipped with a fluorometer, and data were interpolated to 1-m depth intervals. Dynamic heights at the stations were calculated with a reference depth level of 500 m. Where station depth was lower, dynamic height was extrapolated using the continuity equation applied to the deepest level of three-station clusters (cf. Hidaka, 1940). Water samples for chlorophyll-*a* (chl-*a*) determination were collected at some stations using a rosette system at three depths down to 80 m throughout the day and night to calibrate the fluorometer. The chl-*a* concentration (μg/L) was determined fluorometrically (Yentsch and Menzel, 1963). Water samples of 150 mL were filtered through Whatman GF/F filters. Chl-*a* was extracted from filters immersed in 6 mL of 90% acetone (24 h at 4°C in darkness). The extract was analysed with a Turner Designs fluorometer calibrated with pure chl-*a*. The relationship between chl-*a* concentration and fluorescence obtained in each survey was used to convert the continuous CTD fluorescence register into the chl-*a* concentration.

Zooplankton mesoscale sampling was conducted at 35 stations in 2017 and at 29 stations in 2018, using a Bongo net (60 cm diameter and 300 μm mesh size) towed obliquely from a maximum depth of 500 m, or 5 m above the seafloor at shallower stations, to the surface and at a vessel speed of 3.7 km/h (Fig. 1). In addition, depth-stratified samplings were performed at selected stations from a maximum depth of 550 m on the shelf and slope during the day (8:30–18:00 h UTC) and night (20:00–06:00 h UTC), avoiding sunset and sunrise. In 2017, these samplings were performed at 13 stations, three of which were fixed stations sampled for 24 or 48 h, and in 2018 two fixed stations were sampled for 24 h (Fig. 1). Depth-stratified samplings were carried out using a Multiple Opening/Closing Net Environmental Sensing System (a MOCNESS net with a 1 m^2^ mouth opening consisting of 8 nets with a 300 μm mesh size) towed obliquely at a ship speed of 3.7–4.6 km/h. Depth strata were defined according to the maximum depth at each station (shallow stations 120, 100, 80, 60, 40, 30, 20 and 10 m; deep stations 550, 400, 300, 200, 150, 100, 50 and 25 m). The volume of filtered water was recorded by a flowmeter placed in each net mouth. Immediately after collection, the zooplankton samples were fixed in 5% formaldehyde buffered with sodium tetraborate.

### Sample processing and data analysis

In the laboratory, colonies of *P. atlanticum* were sorted from zooplankton samples using a stereomicroscope. All *P. atlanticum* colonies were counted, but when samples were estimated to contain >200 colonies, aliquots were taken to obtain at least 100 colonies and extrapolate the count to the whole sample. To determine colony size, all *P. atlanticum* colonies were scanned using a ZooScan (Hydroptic III) (Grosjean *et al*., 2004) and measured from the diaphragm to the opposite end to the nearest 0.1 mm using the ImageJ software v.1.51j8 (Rasband, 2018). A preliminary exploration of the vertical distribution of *P. atlanticum* colonies (grouped in 1 mm length intervals) revealed that the diel vertical migration did not always initiate at the same colony size but instead was observed in some colonies of 4 mm, some of 5 mm and some of 6 mm (Fig. S1). In addition, colonies of these sizes showed a similar vertical distribution pattern (Fig. S1) and were grouped for the analysis of the vertical distribution. For this analysis, three colony size classes were considered: <4 (including tetrazoids), 4–6.9 and ≥7 mm (hereinafter referred to as small, medium and large, respectively). Data from all MOCNESS stations were considered and the number of colonies per size class collected at each depth stratum was standardized to a number per 1000 m^3^ of filtered water at each depth stratum. The horizontal distribution of small and medium colonies was similar and quite different from that of the large colonies, so for the analysis of the mesoscale distribution two size classes were considered (≤6.9 and ≥7 mm; hereinafter referred to as small-medium and large, respectively). This analysis was conducted from data obtained with the Bongo and MOCNESS nets (except at the fixed stations) (Fig. 1). The number of colonies of each size class collected by the Bongo was standardized to a number per 1000 m^3^ of filtered water. The number of colonies of each size class collected by the MOCNESS at each depth stratum was pooled and divided by the sum of the filtered water at each depth stratum and standardized to a number per 1000 m^3^ of filtered water.

To investigate the effect of environmental variables on the mesoscale horizontal distribution of *P. atlanticum*, the explanatory variables considered were temperature, salinity, density and chl-*a* at 10, 30 and 50 m depth, and bathymetry. Temperature and chl-*a* at 10 m, density at 30 m and bathymetry were selected as independent variables after evaluating collinearity through the Pearson cross-correlation (coefficient < |0.6|). A Generalized Linear Model (GLM) was fitted to assess the effect of the independent oceanographic variables on the mesoscale horizontal distribution of small–medium and large *P. atlanticum* colonies. For large colonies, the best model, based on the Akaike information criterion (AIC) (Akaike, 1974) and residual inspection, showed a structure that was included in a Generalized Linear Mixed Model (GLMM), considering the region of the sampling stations (“shelf” when <200 m depth; and “slope” when >200 m depth) as a random effect. In addition, standardized environmental variables [(value-mean)/standard deviation (SD)] were required for the convergence of the GLMM. The number of *P. atlanticum* colonies (counts) of both size classes, which followed a negative binomial distribution, was analysed using the “glm.nb” (for GLM) and “glmer.nb” function (for GLMM) from the “MASS” package and with a log-link to avoid predicting negative numbers of colonies (Venables and Ripley, 2002). The (log-transformed) volume of filtered seawater was included as an offset inside GLM and GLMM to reduce the bias owing to different volumes filtered by the nets (826 m^3^ mean, 857 SD) (Zuur *et al*., 2009; Canepa *et al*., 2017). In both cases, the optimal model was obtained through a backward selection criterion based on the significance of each explanatory variable (*α* = 0.05), using the AIC comparison and through the inspection of the residuals. Models were carried out using the R statistical programming language v3.5.3 (R Core Team, 2020). Maps of temperature, salinity and chl-*a* at 10 m and density at 30 m for 2017 and 2018 were generated by means of the minimum curvature interpolation method. Vertical sections of temperature, salinity and chl-*a* for both years were obtained using kriging, considering the anisotropy of the water column. All were performed using Surfer® v13.4 (Golden Software, LLC, 2016).

To analyse the vertical distribution of *P. atlanticum* at each size class during day and night, the weighted mean depth (WMD) was calculated for each sampling station (MOCNESS net) by size class and light (day/night) as follows:

$$ \text{WMD}=\sum_{i=1}^n{P}_i{Z}_i $$

where *P_i_* is the proportion of colonies in the *i*th depth stratum:

$$ {P}_i=\frac{C_i{H}_i}{\sum_{i=1}^n{C}_i{H}_i} $$

*Z_i_* is the mean sampling depth of the *i*th depth stratum; *C_i_* is the concentration of colonies in the *i*th depth stratum and *H_i_* is the width of the *i*th depth stratum.

The effect of size class (<4, 4–6.9, ≥7 mm), light (day/night) and different oceanographic variables on the vertical distribution of *P. atlanticum* was tested through a GLM. Mean temperature, salinity, density and chl-*a* concentration were calculated for each depth stratum and MOCNESS haul. After evaluating collinearity through the Pearson cross-correlation test, temperature and chl-*a* were selected as independent variables (coefficient < |0.6|). The vertical counts of *P. atlanticum*, following a negative binomial distribution, were analysed using the “glm.nb” function from the “MASS” package and with a log-link function (Venables and Ripley, 2002; Zuur *et al*., 2009). The (log-transformed) volume of filtered seawater at each depth stratum was included as an offset inside GLM to reduce the bias arising from different volumes filtered by the nets (216 m^3^ mean, 276 SD) (Zuur *et al*., 2009; Canepa *et al*., 2017), and the oceanographic variables were standardized. The GLM was carried out using the R statistical programming language v3.5.3 (R Core Team, 2020).

## RESULTS

### Hydrographic conditions

**Fig. 2:**
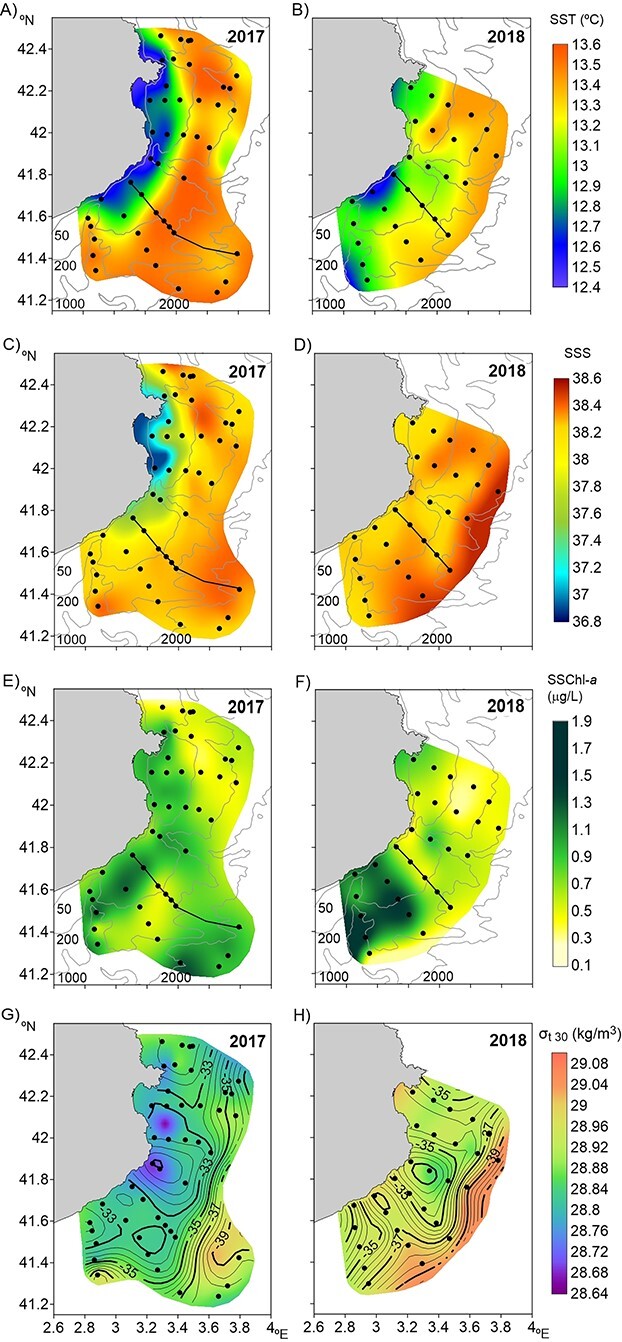
Maps of surface (10 m) temperature (SST; **A**, **B**), salinity (SSS; **C**, **D**) and chlorophyll-*a* (SSChl-*a*; **E**, **F**) and dynamic height (contour lines, dynamic cm) at 30 m relative to 500 m overlaid on density at 30 m (*σ*_t 30_; **G**, **H**) in winter 2017 (left panels) and 2018 (right panels). Thin grey lines show isobaths (50, 200, 1000 and 2000 m).

**Fig. 3:**
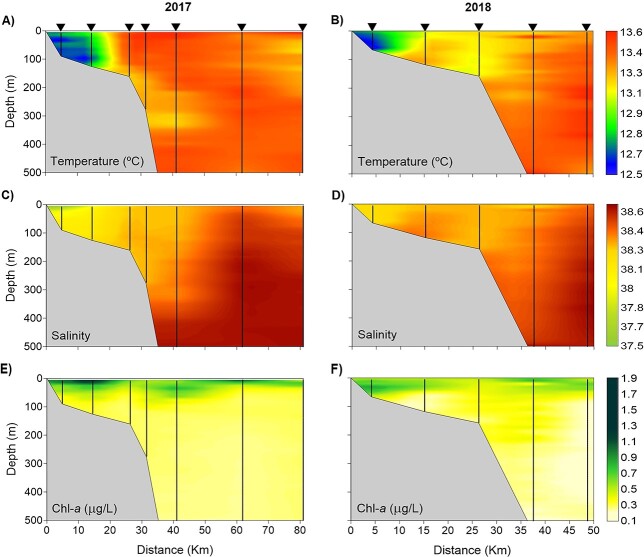
Vertical distribution of temperature (**A**, **B**), salinity (**C**, **D**) and chlorophyll-*a* (**E**, **F**) in the upper 500 m depth in winter 2017 (left panels) and 2018 (right panels) in the sections marked in Fig. 1. Horizontal axis indicates distance from the coast. Vertical lines represent 1 m binned CTD profile data.

In both years, surface temperature and salinity increased towards the open sea (Fig. 2**A**–**D**). In 2017, the coastal zone showed low temperature (≈12.5°C) and salinity (≈37.2), whereas on the shelf edge and slope, the temperature reached 13.6°C and salinity 38.4. In 2018, coastal waters also showed low temperature (≈12.9°C) and relatively low salinity (≈38.3); on the shelf edge and in the open sea, salinity was similar to that detected in the previous year and temperature was slightly lower, around 13.2°C. Surface chl-*a* did not show a consistent pattern (Fig. 2**E** and **F**). In 2017, relatively high values were detected on the shelf, but also in the open sea (≈1.2 μg/L), whereas in 2018, high chl-*a* concentrations (≈1.9 μg/L) were found in the southwest part of the area. On both cruises, the distribution of density at 30 m increased from near the coast towards the open sea, following a similar pattern to that of salinity. The dynamic height overlaid on the density at 30 m showed the signature of the shelf-slope front associated with the Northern Current (Font *et al*., 1988) along the continental slope (Fig. 2**G** and **H**). Some detected intrusions onto the shelf were related to the instabilities of the current (Fig. 2G and H). The vertical section of temperature and salinity showed the presence of colder and less saline waters close to the coast, which reached the seafloor up to around 100 m depth in both years and were more evident in 2017 (Fig. **3A**–**D**). Chl-*a* was detected in the first 80 m of the water column, with the highest values in the upper ≈25 m (≈1.15 μg/L in 2017 and ≈0.80 μg/L in 2018) (Fig. 3**E** and **F**).

### Horizontal distribution of *P. atlanctium*

**Fig. 4:**
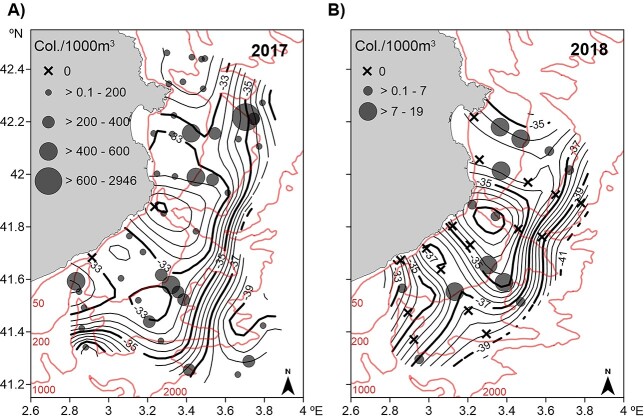
Horizontal distribution of small-medium (≤6.9 mm) *Pyrosoma atlanticum* colonies overlaid on dynamic height (contour lines; dynamic cm) at 30 m relative to 500 m and bathymetry (in red; 50, 200, 1000 and 2000 m) in 2017 (**A**) and 2018 (**B**).

**Fig. 5:**
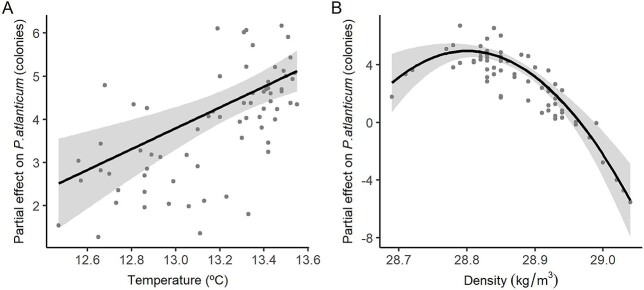
Partial effect of temperature (10 m; **A**) and density (30 m; **B**) on small–medium (≤ 6.9 mm) *Pyrosoma atlanticum* colonies (number). Partial effect shows the change in response variable for each value of the variable on the *x*-axis, holding all other variables constant (median). Central (bold) lines show the best fit, the shaded areas show the 95% confidence intervals of the GLM model and dots correspond to observations.

**Fig. 6:**
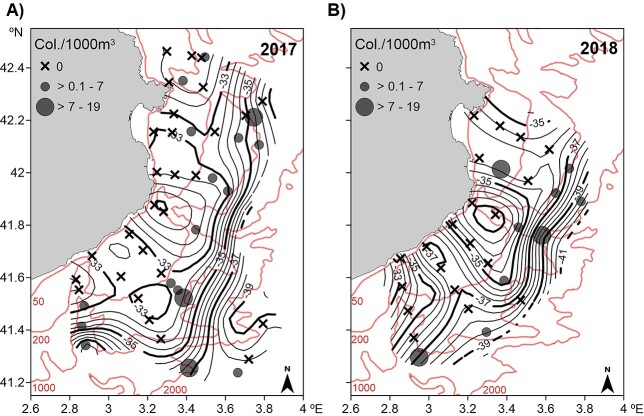
Horizontal distribution of large (≥7 mm) *Pyrosoma atlanticum* colonies overlaid on dynamic height (contour lines; dynamic cm) at 30 m relative to 500 m and bathymetry (in red; 50, 200, 1000 and 2000 m) in 2017 (**A**) and 2018 (**B**).

**Fig. 7:**
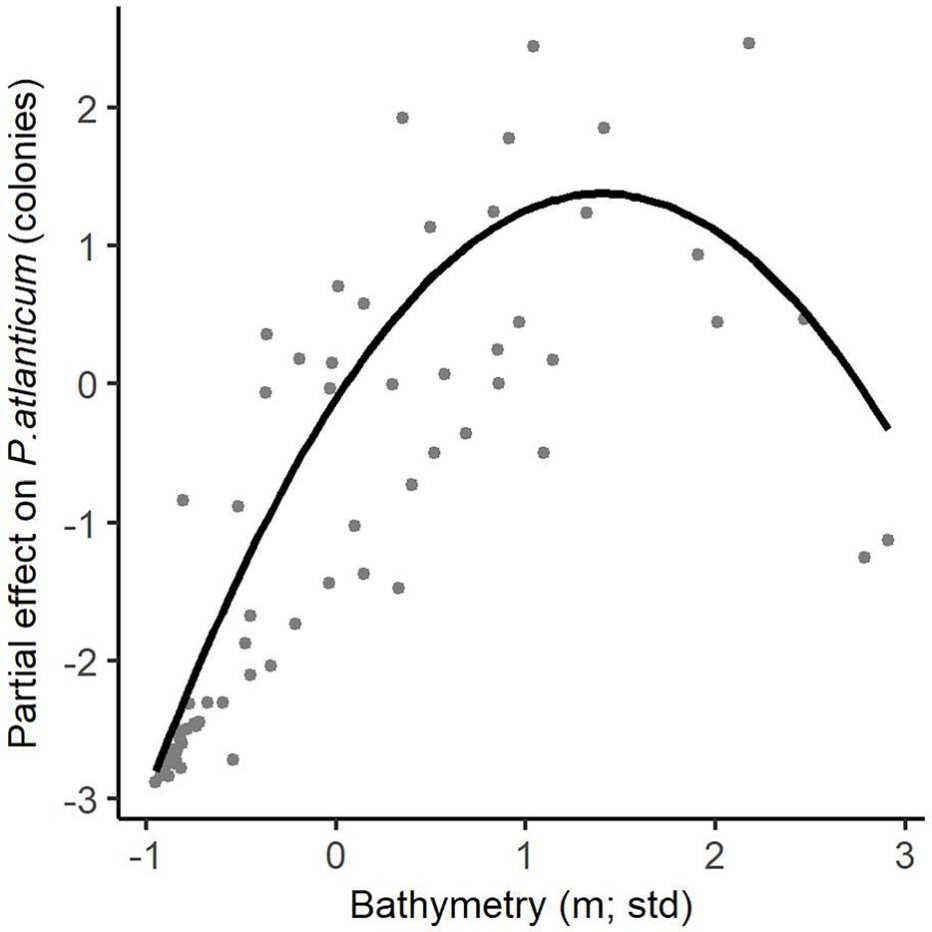
Partial effect of bathymetry (standardized) on large (≥ 7 mm) *Pyrosoma atlanticum* colonies (number). Partial effect shows the change in response variable for each value of the variable on the *x*-axis, holding all other variables constant (median). Bold line show the best fit of the GLMM model and dots correspond to observations.

*P. atlanticum* colonies were much more abundant in 2017 than in 2018 (mean abundance 228.70 col./1000 m^3^, 394.77 SD and 5.12 col./1000 m^3^, 6.08 SD, respectively), owing to the massive concentration of small size colonies in 2017. However, small colonies (<4 mm) dominated in both years, 2–3 mm (range: 0.9–82.0 mm) in 2017 and 1–2 mm (range: 1.2–59.0 mm) in 2018 (Fig. S2). The distribution pattern of colonies within the small–medium (≤6.9 mm) and large (≥7 mm) size classes was quite similar in both years. The small–medium colonies were present all over the area, with low abundances or even absence at stations close to the coast and high abundances on the shelf edge (Fig. 4**A** and **B**). The GLM identified surface temperature and density (30 m) as significant variables (*P* < 0.05) related to *P. atlanticum* distribution (Table I, Fig. S3). High abundances were found in warm waters (13.1–13.6°C) and of moderate density (28.7–28.9 kg/m^3^) (Fig. 5**A** and **B**). Large colonies were mainly found on the slope at depths greater than 400 m and in relatively low chl-*a* surface waters (0.5–1.0 μg/L) (Figs 2E and F and 6**A** and **B**) in the vicinity of the front throughout the area, but were practically absent in shelf waters. The GLMM identified bathymetry as a significant variable (*P* < 0.05) related to this size class distribution (Table I, Fig. 7, and Fig. S4). Although surface chl-*a* was not a significant variable for the distribution of large colonies (negative relationship, Table I, Fig. S5), it was kept in the GLMM because its inclusion improved the explanatory capacity of the model, providing a lower AIC value (Fig. S4) (Zuur *et al*., 2009).

**Table I:** *Model results for horizontal distribution of small-medium colonies (≤6.9 mm; GLM) and large colonies (≥7 mm; GLMM) of Pyrosoma atlanticum.* ^*^ α = 0.05; std = standardized variables; n.s. = non-significant; + variable still included in the model following AIC criteria

Model	Parameter	Estimate	*z*-value	*P*-value
**GLM**	Intercept	−1.50e^5^	−4.95	< 0.01^*^
	Temperature (10 m)	2.41	3.95	< 0.01^*^
	Density (30 m)	1.04e^4^	4.96	< 0.01^*^
	Density (30 m) ^2^	−1.81e^2^	−4.97	< 0.01^*^
**GLMM**	Intercept	−6.85	−22.22	< 0.01^*^
	Bathymetry (std)	2.12	4.22	< 0.01^*^
	Bathymetry (std)^2^	−0.76	−3.01	< 0.01^*^
	Chlorophyll-*a* (10 m) (std)	−0.54	−1.64	0.101 n.s.^+^

### Vertical distribution of *P. atlanticum*

**Fig. 8:**
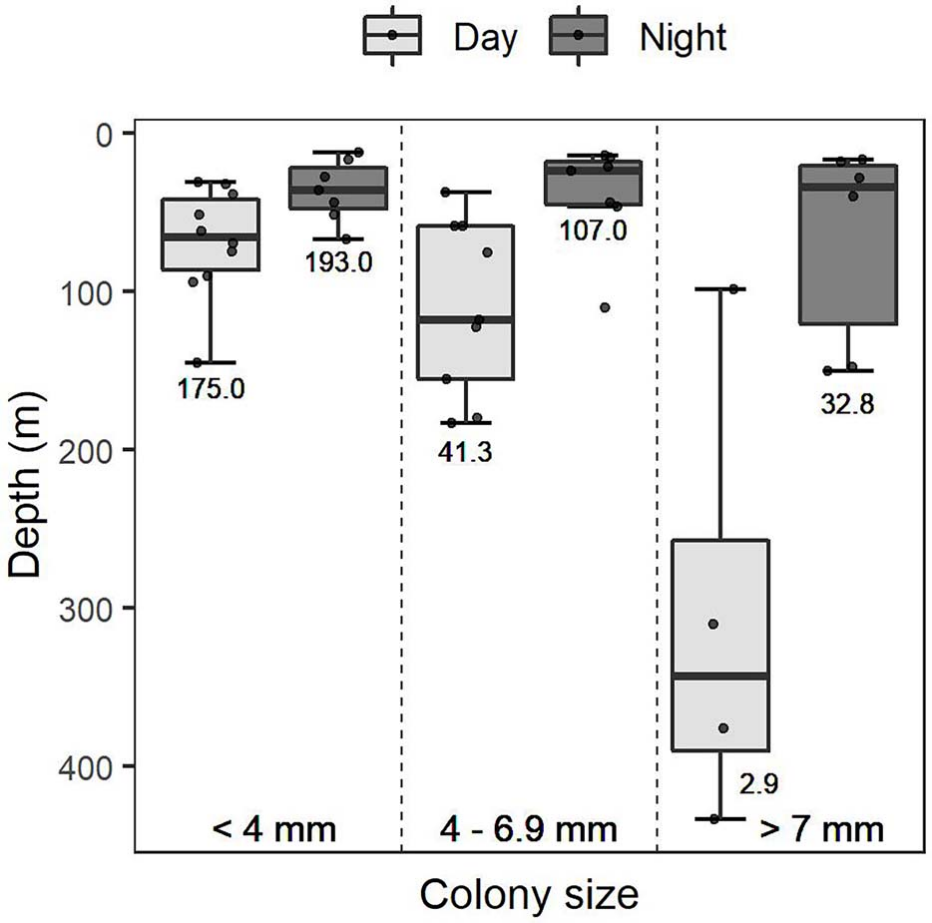
Weighted mean depth (WMD) of *Pyrosoma atlanticum* by colony size class [<4 mm (small), 4–6.9 mm (medium) and ≥7 mm (large)] during day-time (light grey) and night-time (dark grey). The central marks of each box represent the median of the WMD, the boxes show the interquartile ranges and the whiskers correspond to the ranges of observations. Mean abundance of colonies is indicated below each box (col./1000 m^3^).

**Fig. 9:**
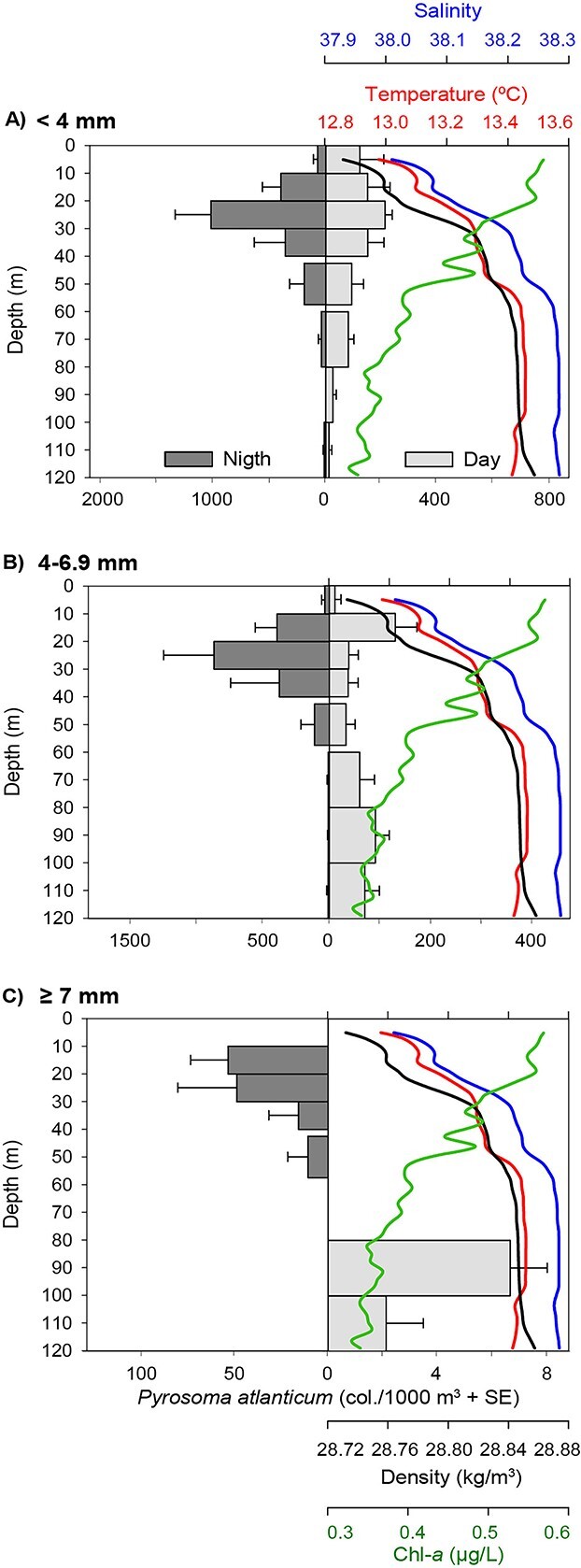
Mean vertical distribution (+SE) of *Pyrosoma atlanticum* by colony size class [<4 mm (small), **A**; 4–6.9 mm (medium), **B**; ≥7 mm (large), **C**] in night-time (dark grey bars) and day-time (light grey bars), overlaid on vertical profiles of temperature (red), salinity (blue), density (black) and chlorophyll-*a* (green). Note that the colony abundance scales are different for each size class. Data correspond to the 48 h fixed station sampled in 2017 (Fig. 1).

The GLM analysis indicated a significant effect (*P* < 0.01) of the colony size class (<4, 4–6.9, ≥7 mm), light level (day/night) and mean chl-*a* concentration by sampling strata on *P. atlanticum* vertical distribution (Table II). During the night, colonies of all size classes were located in the upper part of the water column (WMD was 36, 39 and 67 m for the small, medium and large colonies, respectively; Table III), whereas during the day their distribution varied significantly depending on the colony size. Thus, during daylight hours, small colonies remained in the upper part of the water column (WMD = 69 m), medium size colonies showed a slightly deeper distribution (WMD = 110 m) with a migration amplitude of 71 m and large colonies were located deeper (WMD = 304 m), with a migration amplitude of 237 m (Fig. 8, Table III), reaching a maximum depth of 550 m. The fine-scale vertical distribution obtained at the fixed station performed in 2017 (Fig. 1) followed the pattern described above and allowed us to visualize its relation to the chl-*a* concentration in the water column (Fig. 9). Small colonies remained, day and night, in the upper part of the water column (mainly between 10 and 40 m), coinciding with a high chl-*a* concentration in these waters. Medium colonies were also located between 10 and 40 m at night, whereas during the day they showed a wider distribution (between 10 and 120 m), around 52% of the colonies migrating downwards and the rest remaining in the upper layers (Fig. 9). This distribution pattern was followed by colonies of each millimetre (4, 5 and 6 mm) within this medium size class (Fig. S1). Large colonies showed a clearly different distribution between day and night, being located in shallow (10–30 m) productive waters during the night and in deeper waters (below 80 m) during the day (Fig. 9). Overall, the abundance of colonies was higher during the night than during the day, and this pattern was more marked in the largest size class (Figs 8 and 9).

**Table II:** *GLM results for chlorophyll-a, colony size class [<4 mm (small), 4–6.9 mm (medium) and ≥7 mm (large)] and light (day-time/night-time) effects on Pyrosoma atlanticum vertical distribution*. ^^*^^α = 0.05; std = standardized variable

Parameter	Deviance	Pr (>Chi)
**Mean chl-*a* (std)**	102.52	< 0.01^*^
**Colony size**	59.60	< 0.01^*^
**Light (day/night)**	8.92	< 0.01^*^
**Colony size: light (day/night)**	16.16	< 0.01^*^

**Table III:** Average weighted mean depth (WMD) and standard deviation (SD) during day-time and night-time and migration amplitude of Pyrosoma atlanticum by colony size class

Size class	WMD (m)				Migration amplitude (m)
	Day		Night		
	Mean	SD	Mean	SD	
<4 mm (small)	69	35	36	19	33
4–6.9 mm (medium)	110	55	39	34	71
≥7 mm (large)	304	146	67	64	237

## DISCUSSION

Our study evidenced that the spatial distribution (horizontal and vertical) of *P. atlanticum* in the Mediterranean Sea depends on the colony size and is ultimately determined by oceanographic and biological structures. The abundance and size range of *P. atlanticum* colonies found in our study were similar to those recorded previously in the NW Mediterranean: maximum abundance of 7 col./1000 m^3^, 8–88 mm long (Andersen *et al*., 1992); max. 187 col./1000 m^3^, 3–51 mm (Andersen and Sardou, 1994); max. 2000 col./1000 m^3^, 20 mm (Braconnot and Goy, 1981); max. 900 col./1000 m^3^, 4–6 mm and max. 213 col./1000 m^3^, 50 mm long (Granata *et al*., 2020). In the Atlantic and Pacific oceans, the abundances reported using a similar sampling methodology were much higher and colonies were generally larger, reaching 41 000 col./1000 m^3^ for colonies between 50 and 65 mm long (Drits *et al*., 1992) and 5000 col./1000 m^3^ for colonies between 60 and 780 mm long (Schram *et al*., 2020). In both years studied, the small size classes were dominant (Fig. S2), suggesting that sexual reproduction occurs in winter (Franqueville, 1971; Braconnot, 1974). Several studies carried out in the Mediterranean and northeast Pacific also reported a high abundance of small colonies in late winter and spring (Franqueville, 1971; O’Loughlin *et al*., 2020; Lyle *et al*., 2022). Although in both years the sampling was conducted in February, in 2017, the abundance of small–medium (≤6.9 mm) colonies was very high, the largest registered in the Mediterranean (Andersen and Sardou, 1994; Granata *et al*., 2020). This exceptional abundance might be related to an earlier development of spring conditions and the subsequent seasonal phytoplankton bloom in 2017 in comparison with 2018 (Mir-Arguimbau *et al*., 2022). The life history traits of *P. atlanticum*, with short generation times and rapid growth, could allow for a rapid population increase during suitable trophic conditions associated with the seasonal production bloom (Alldredge and Madin, 1982).

### Horizontal distribution of *P. atlanticum*

The mesoscale distribution of *P. atlanticum* showed that large (≥7 mm) colonies were found on the slope (around 400 m depth), coinciding with the presence of the front all along the area (Fig. 6). This suggests that the species inhabits water over the slope, and the front could aggregate and prevent its dispersion towards the open sea, as reported in other zooplanktonic organisms in the area (Masó *et al*., 1998; Guerrero *et al*., 2016). However, the lack of sampling beyond the front precludes any knowledge of the abundance and distribution of *P. atlanticum* in open sea waters, where the species has also been found in the Mediterranean (Bo *et al*., 2020; Granata *et al*., 2020). Although it is a filter feeder, a negative but non-significant relationship was found between colony abundance and chl-*a* concentration (Figs 2E and F and 6, Fig. S5). It has been reported that high abundances of phytoplankton prey, usually found in coastal waters, may become harmful because the mucous filters of *P. atlanticum* can become clogged (Harbison *et al*., 1986; Lyle *et al*., 2022). Also, it has been suggested that food quality rather than its availability is a determining factor in the species distribution (Schram *et al*., 2020). However, Henschke *et al*. (2019) reported that chl-*a* concentration was a significant driver of *P. atlanticum* biomass.

In both years studied, small–medium colonies were present throughout the area, with the highest abundances along the edge of the shelf, being very low close to the coast, where the waters were colder and less saline (Figs 2A–D and 4). However, the presence of *P. atlanticum* in colder (7–10°C) (Thompson, 1948; Sutherland *et al*., 2018) and less saline (31–33) waters than the Mediterranean (Schram *et al*., 2020) suggests that the temperature and salinity values detected near the coast do not represent a limitation to its distribution, and the low abundance of *P. atlanticum* in the coastal area is probably associated with the intrusion of a coastal water mass. A high abundance of small-medium colonies was found at the moderate water density values that were found at the shelf edge, close to the highest abundance of large colonies on the slope (Fig. 2G, H, 4, 6). This distribution would support the idea that the young colonies would be offspring of the large ones, taking into account that sexually mature zooids appear in colonies of 40 mm onwards (Van Soest, 1981) and that young colonies grow rapidly (coefficients of exponential growth of 0.24–0.75 per day on a length basis (Andersen and Sardou, 1994)). Overall, the observed distributions suggest that large *P. atlanticum* inhabit waters over the slope, where reproduction might take place, in association with the front, and the presence of young colonies on the shelf could be related to the offshore water intrusions associated with instabilities of the front (Sabatés *et al*., 2004). Similar observations have been made in this area for other gelatinous organisms. Sabatés *et al*. (2018) reported that adult stages of the Scyphozoan *Pelagia noctiluca* were found along the slope in association with the front. Ephyrae (young stages), which inhabit surface waters (Ottmann *et al*., 2021; Pastor-Prieto *et al*., 2021), showed a wider distribution extending over the shelf and their occurrence was associated with offshore water intrusions generated by the oscillatory behaviour of the front. The frontal area is a transitional zone with enhanced primary and secondary production (Estrada and Margalef, 1988; Ibanez and Boucher, 1987; Sabatés *et al*., 2004) that offers favourable feeding conditions for the reproduction, growth and survival of gelatinous organisms. Nevertheless, it is possible that *P. atlanticum* colonies, as planktonic organisms with limited horizontal mobility, were passively accumulated in that area by physical discontinuities of the ocean, such as fronts and pycnoclines (Graham *et al*., 2001; Greer *et al*., 2015, 2018). Studies conducted in the Atlantic and Pacific oceans have suggested that *P. atlanticum* colonies were located in open sea waters (Angel, 1989; Brodeur *et al*., 2018) and that they may have been transported by advection to the shelf, where they were less abundant (Miller *et al*., 2019; Schram *et al*., 2020; Lyle *et al*., 2022).

### Vertical distribution of *P. atlanticum*

The analysis of the vertical distribution of *P. atlanticum* allowed us to detect different migration amplitudes as a function of colony size (Fig. 8). Small colonies showed no diel vertical migration, being mainly located in the upper 40 m of the water column during both day and night, as previously observed by Palma (1985) in the Mediterranean (Fig. 9). The migratory behaviour of remaining in the surface layers during the night and going to deeper layers during the day started in some colonies of 4, 5 and 6 mm (the medium size class), but not all colonies of these sizes exhibited this behaviour (Fig. S1). This would suggest that migration, as an individual response, can start at any of these sizes. The migration amplitude increased from 71 m in medium colonies to 237 m in the large ones. A similar migration amplitude (210 m) for the same size colonies was reported by Sardou *et al*. (1996) in the Mediterranean, whereas other studies described more extensive migrations in that area (515 m) (Andersen *et al*., 1992) and in the Atlantic Ocean (650 m) (Roe *et al*., 1987; Angel, 1989; Andersen *et al*., 1992). Considering that *P. atlanticum* is a strong vertical migrator, being able to reach depths of up to 900 m in the Mediterranean (Sardou *et al*., 1996) and 2500 m in the Atlantic (Roe *et al*., 1987), the limited maximum depth reached in our study (550 m) might be related to the lower depth of our samplings (maximum 550 m). The lower abundance of colonies during the day than during the night, particularly in the largest size class, could be due to the location of colonies below the sampling depth (Fig. 8).

In this study, the lack of stratification typical of winter conditions allowed us to observe the migratory behaviour of the species without the presence of clines that could influence their vertical movement (Graham *et al*., 2001). Peak densities of colonies had been associated with vertical gradients of environmental parameters such as density and fluorescence (Lyle *et al*., 2022). The shallow levels reached by medium and large colonies during the night correspond to the highest values of chl-*a* in the water column (Fig. 9), a proxy of photosynthetic taxa, the main prey of the species (Drits *et al*., 1992; Perissinotto *et al*., 2007). *P. atlanticum* mainly feeds on a wide variety of phytoplankton [e.g. diatoms, dinoflagellates, prymnesiophytes and coccolithophores (Drits *et al*., 1992; Perissinotto *et al*., 2007)] by continuous filtration of seawater (Alldredge and Madin, 1982). Thus, colonies would be expected to find higher food availability in surface waters than in deeper ones. On the other hand, pelagic tunicates also exhibit high filtration rates of microbial prey, including heterotrophic bacteria (Sutherland *et al*., 2010; Sutherland and Thompson, 2021; Thompson *et al*., 2021), which probably allow them to feed at depth and supplement the food acquired in surface waters. The vertical migration pattern observed in *P. atlanticum* is that followed by most zooplankton, which ascend to upper layers during the night to take advantage of the high phytoplankton abundance at the surface before returning to deeper layers during the day (Lampert, 1989; Saiz *et al*., 2014) to avoid predation in the illuminated layers (Bollens and Frost, 1989). However, other factors such as reproductive behaviour may also play a role, with adults migrating towards the surface to provide a suitable environment for the development of their offspring (Lampert, 1989; Ferraris *et al*., 2012). The location of small colonies both day and night in the upper layers, where chl-*a* levels are highest, suggests a strategy of maximizing the colony growth to reduce the high predation rates of small size organisms (Miller *et al*., 1988). Ontogenetic variations in the vertical distribution are a common trait of other zooplanktonic taxa, such as copepods (Andersen *et al*., 2001) and euphausiids (Pillar *et al*., 1989), with younger stages inhabiting shallower waters and adults performing the typical diel vertical migration pattern. It should also be considered that the permanency of small colonies in surface waters may be related to their limited migratory capacity, since their propulsive capacity—and hence migratory amplitude—increase with colony size. The growth of the colony would not only increase its propulsive capacity, but also enhance its visibility, forcing the colony to migrate deeper to avoid visual predators (Angel, 1979). It is unclear whether the increase in migration amplitude with colony size is due to higher visibility, increased propulsive capacity, or a combination of the two.

### Ecological implications

The vertical migration performed by *P. atlanticum* might enhance the vertical transport of carbon to deeper waters. The species shows one of the highest clearance rates of any zooplankton grazer (Perissinotto *et al*., 2007), rapidly producing a high amount of faecal pellets (Drits *et al*., 1992) that are transported to deep waters through diel vertical migration (Henschke *et al*., 2019). It has been reported that mass deposition of *P. atlanticum* may provide an extra input of carbon to benthic consumers (Carrassón and Cartes, 2002; Lebrato and Jones, 2009; Lebrato *et al*., 2013), which rely on the contribution of nutrients from the surface (Smetacek, 1984). In bloom conditions, *P. atlanticum* can exert considerable control over phytoplankton standing stocks (Drits *et al*., 1992; O’Loughlin *et al*., 2020) through competition or direct grazing, playing an important role in the marine food web dynamics (Andersen, 1998; Lavaniegos and Ohman, 2003). Although several *P. atlanticum* bloom events have been reported worldwide, the most impressive was detected in the northeast Pacific, with high abundances of large colonies lasting several years, disrupting marine activities and altering the ecosystem (Brodeur *et al*., 2018, 2019; Schram *et al*., 2020). In the NW Mediterranean, the smaller colonies (Andersen *et al*., 1992; Andersen and Sardou, 1994; this study) and weaker blooms of *P. atlanticum* and other filter-feeding gelatinous taxa than in other regions (Andersen, 1998; Granata *et al*., 2020; O’Loughlin *et al*., 2020) could be related to the oligotrophic nature of this sea. Following Henschke *et al*. (2019), we have estimated that downward carbon transport in our study area would be around 5.26 μg C/m^3^ day (0.56 mg C/m^2^ day) in 2017 and 0.54 μg C/m^3^ day (0.05 mg C/m^2^ day) in 2018 (defecation contributing to ≈17% and ≈14%, respectively; see Supplementary Material). These values would be at the lower limit of the estimated ranges of downward carbon transport (0.42–59.57 mg C/m^2^ day) by mesozooplankton and macrozooplankton communities in the Mediterranean (Frangoulis *et al*., 2011; Isla *et al*., 2015; Yebra *et al*., 2018). Compared with previous estimates for *P. atlanticum*, our obtained values were two and three orders of magnitude lower than the 363 μg C/m^3^ day reported in the Tasman Sea (Henschke *et al*., 2019). However, considering the chl-*a* (i.e. carbon) concentration in the upper water layers observed in both regions (1.33 μg chl-*a*/L in the Tasman Sea, 0.65 μg chl-*a*/L in 2017 and 0.86 μg chl-*a*/L in 2018 in our study area), these differences decrease by one order of magnitude each year (see Supplementary Material). However, the lower values estimated in the present study are probably related to the smaller size of colonies than those observed in the Tasman Sea (range: 11–318 mm) (Henschke *et al*., 2019).

Although our results show a strong difference in the abundance of *P. atlanticum* in the two years studied, longer time series would be necessary to confirm the high interannual variability that has been described for gelatinous zooplankton in the Mediterranean (García-Comas *et al*., 2011;Fullgrabe *et al*., 2020;Feuilloley *et al*., 2021). This high variability has been related to the winter environmental conditions, which cause mixing of the water column and the input of nutrients to the surface waters, ultimately modulating the phytoplankton bloom (García-Comas *et al*., 2011; Fullgrabe *et al*., 2020). The recently observed climatic trends for the Mediterranean, showing an increase in sea water temperature, a lower wind speed and a lengthening of the seasonal stratification period (Rixen *et al*., 2005; Calvo *et al*., 2011; Vargas-Yáñez *et al*., 2017), could modify the intensity and regularity of phytoplankton blooms, altering the abundance, distribution and species composition of gelatinous zooplankton (Guerrero *et al*., 2018a). Future studies should address the long-term interannual variability of *P. atlanticum*, as well as relevant aspects of its biology (e.g. feeding and propulsive capacity) to understand its ecological role in the current context of climate change in the Mediterranean.

## CONCLUSIONS

The mesoscale and vertical distribution of *P. atlanticum* in the NW Mediterranean were shaped by the oceanographic and biological structures, as well as by the ontogenetic stage of the colonies. The higher abundance of colonies in 2017 than in 2018 was likely related to an earlier onset of the phytoplankton bloom. Large colonies (≥7 mm) were found on the slope, in association with the shelf-slope front, which would aggregate them, preventing their dispersion towards the open sea. Small (<4 mm) and medium (4–6.9 mm) colonies extended their distribution over the shelf owing to instabilities of the front, and were practically absent in the cold, low-salinity coastal waters. The vertical migration amplitude increased with colony size. At night colonies of all sizes remained close to the surface, where chl-*a* levels were high, whereas during the day they migrated to deeper layers, reaching greater depths as colony size increased. The migratory behaviour started when colonies were 4–6.9 mm long, though not all colonies of these sizes exhibited this behaviour, suggesting that migration can start at any of these sizes. This vertical migration might contribute to carbon transport to depth. Our observations shed light on these gelatinous organisms, scarcely studied in the Mediterranean, which may play a relevant role in the marine trophic web.

## Supplementary Material

RevisedSupplementaryMaterial_fbac056
