# Supplementary material for: The role of oceanographic conditions and colony size in shaping the spatial structure of Pyrosoma atlanticum in the NW Mediterranean Sea
Source: J Plankton Res. 2022 Oct 12;44(6):984–99. doi: 10.1093/plankt/fbac056 (PMC9692197; doi:10.1093/plankt/fbac056)
Supplement: RevisedSupplementaryMaterial_fbac056 [file revisedsupplementarymaterial_fbac056.docx]

**Supplementary material**


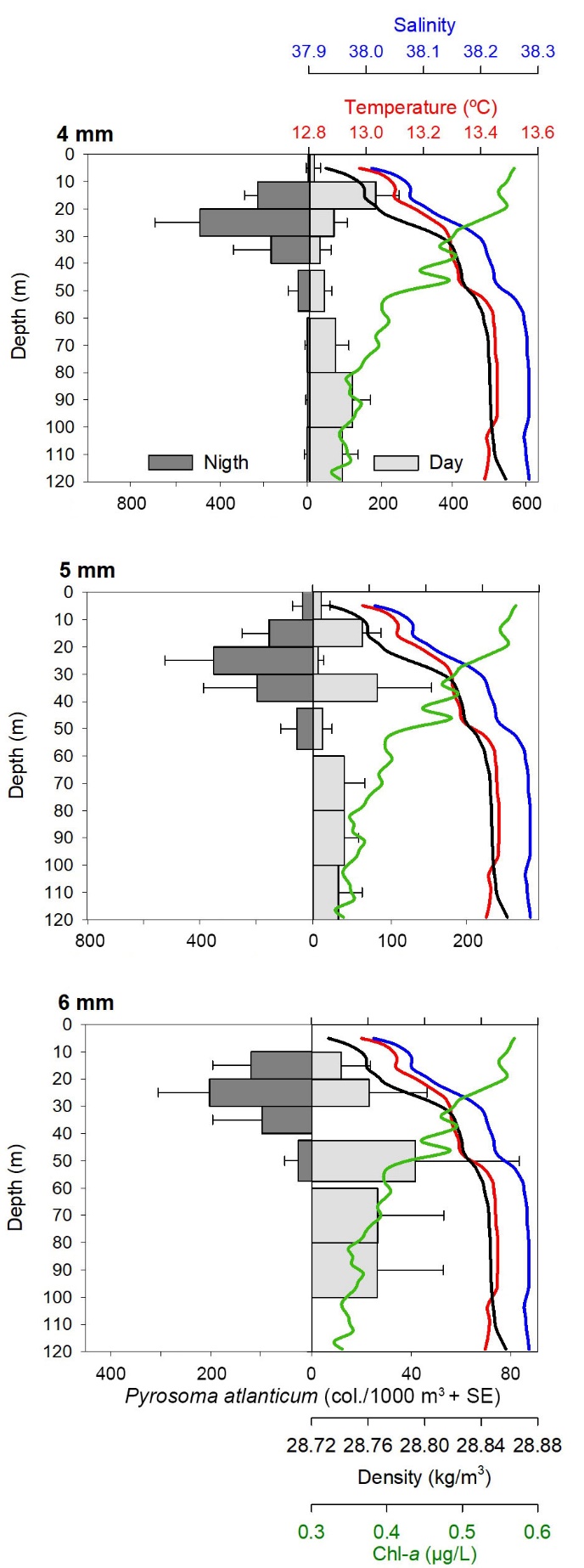


**Fig. S1:** Mean vertical distribution (+ standard error) of *Pyrosoma atlanticum* by millimetre within the medium size class (4-6.9 mm), in night-time (dark grey bars) and day-time (light grey bars), overlaid on vertical profiles of temperature (red), salinity (blue), density (black) and chlorophyll-*a* (green). Note that the colony abundance scales are different for each colony size. Data correspond to the 48h fixed station sampled in 2017 (see Fig. 1).

**
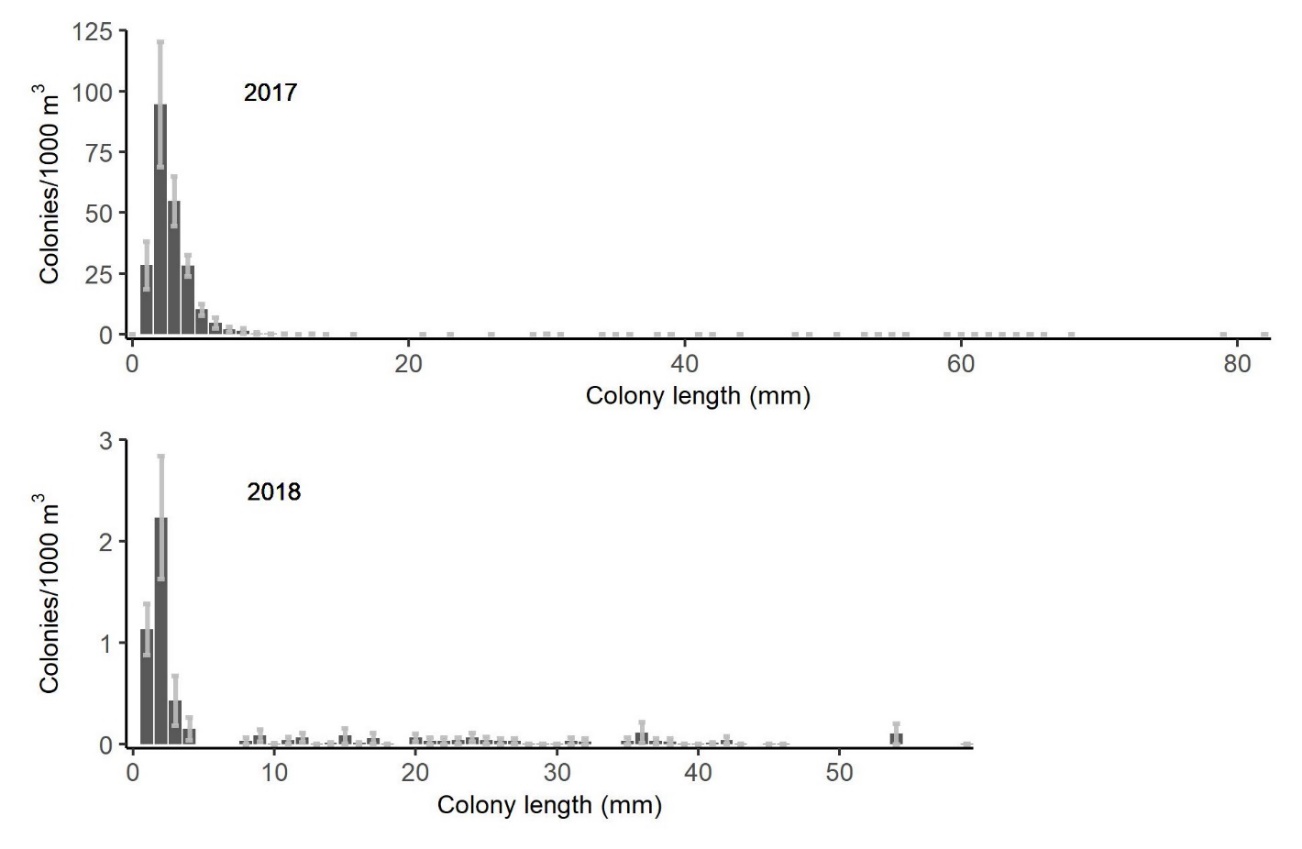
Fig. S2:** Size frequency distribution (mean ± standard error) of *Pyrosoma atlanticum* colonies in 2017 (upper panel) and 2018 (lower panel). Note the different axis for colony abundance in each year.

**
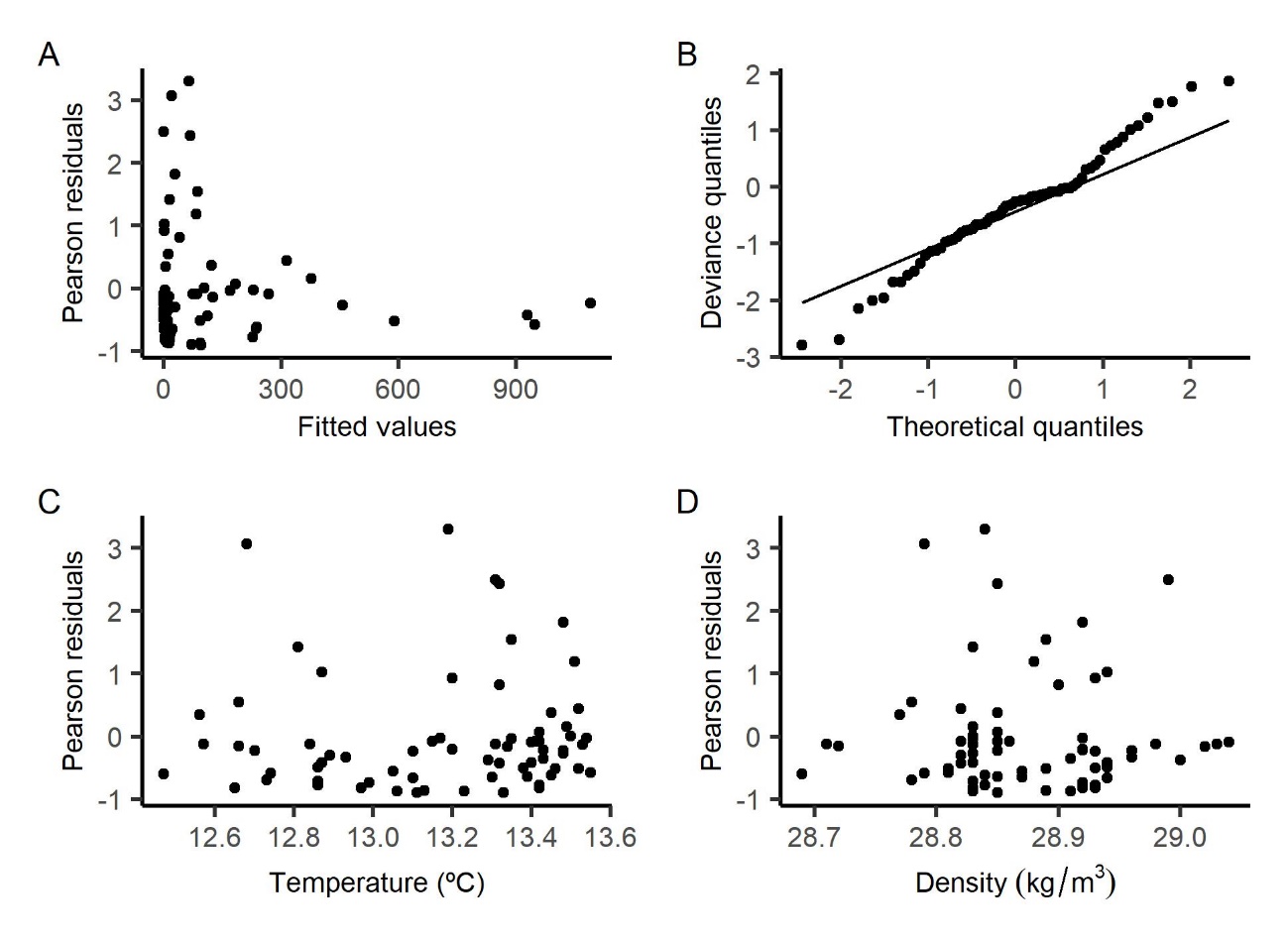
**

**Fig. S3:** Residual diagnostics of GLM analysis on the effect of temperature and density on the horizontal distribution of small-medium (≤ 6.9 mm) colonies of Pyrosoma atlanticum. Residuals distribution vs. fitted values (a); Q-Q plot (b); residuals distribution vs. temperature (10 m; ºC) (c); residuals distribution vs. density (30 m; kg/m^3^) (d).


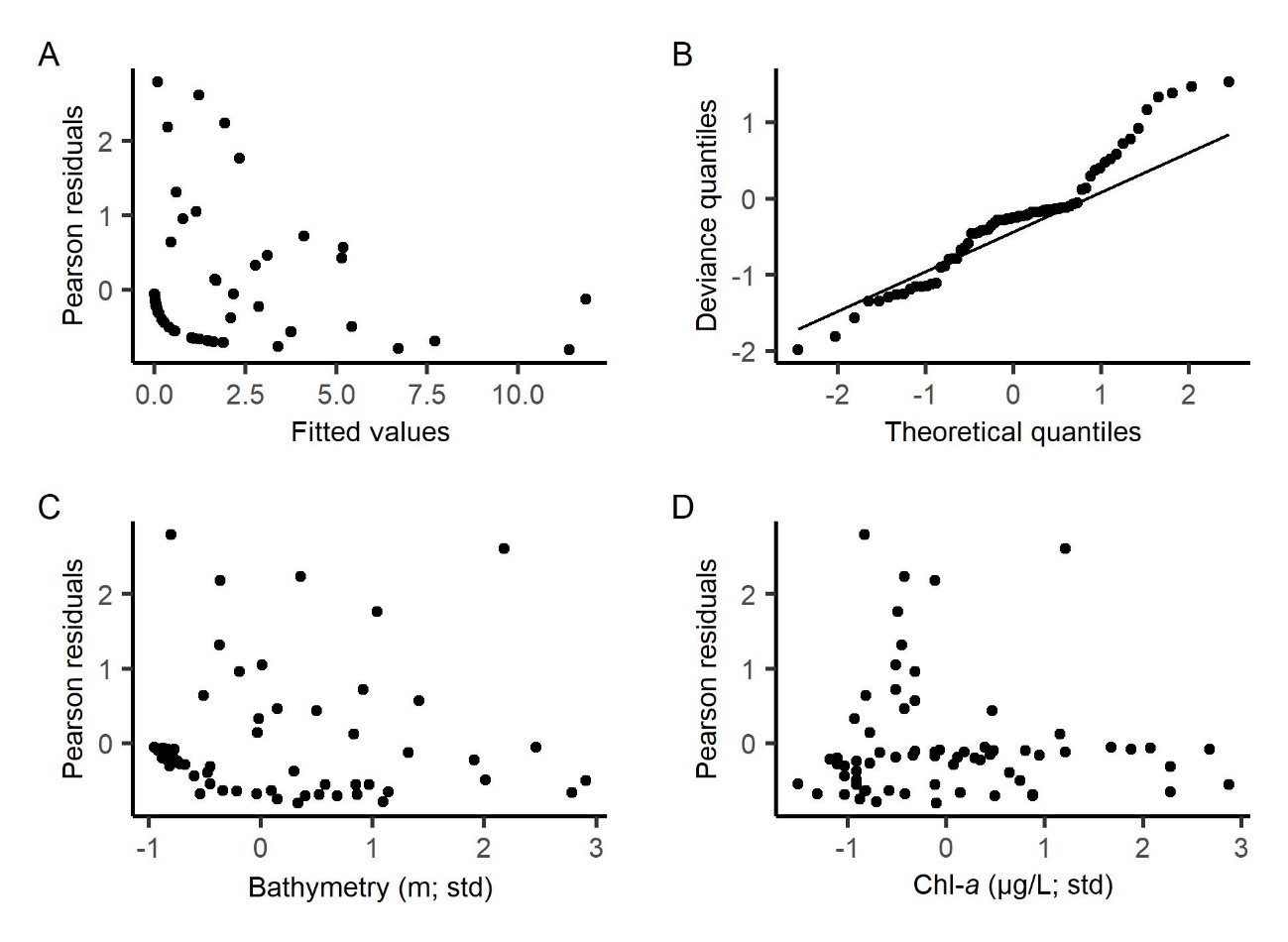


**Fig. S4:** Residual diagnostics of GLMM analysis on the effect of bathymetry and chlorophyll-*a* on the horizontal distribution of large (≥ 7 mm) colonies of Pyrosoma atlanticum. Residuals distribution vs. fitted values (a); Q-Q plot (b); residuals distribution vs. bathymetry (m) (c); residuals distribution vs. chlorophyll-*a* (10 m; µg/L) (d). std = standardized variables.


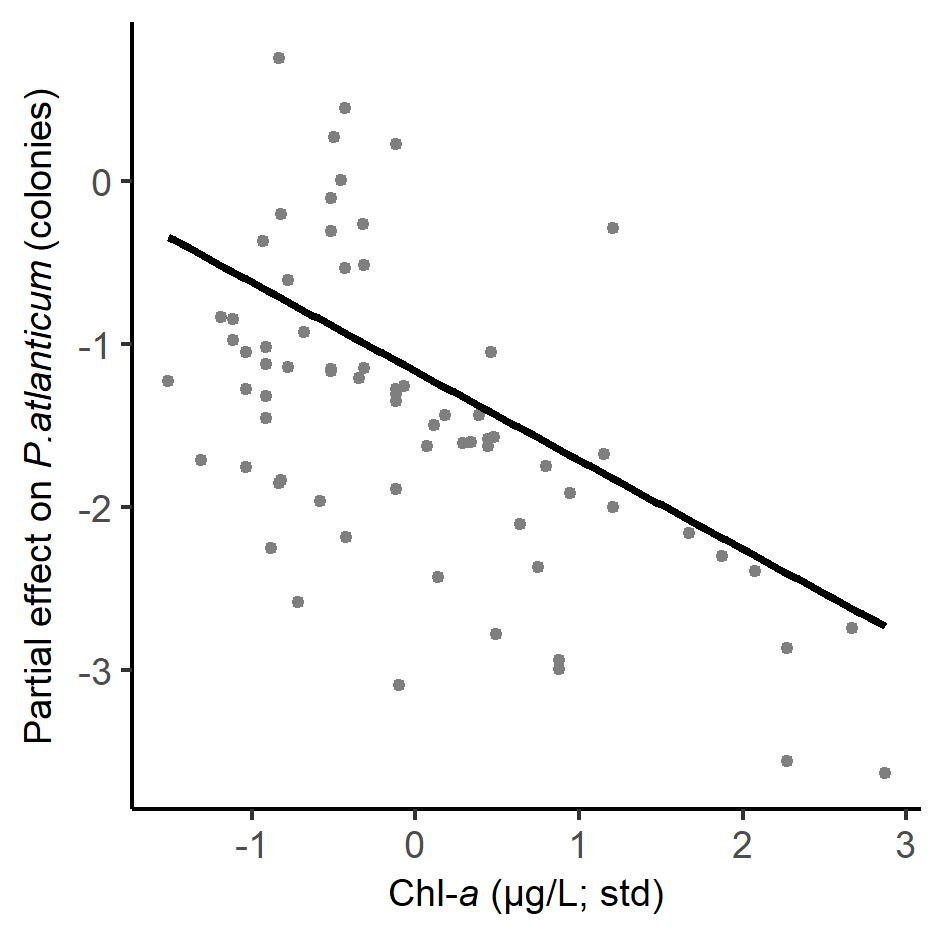


**Fig. S5:** Partial effect of surface (10 m) chlorophyll-*a* (standardized; non significant) over the large *Pyrosoma atlanticum* colonies (number). Partial effect shows the change in response variable for each value of the variable on the x-axis, holding all other variables constant (median). Bold line show the best fit of the GLMM model and dots correspond to observations.

**Estimation of downward active carbon transport by *Pyrosoma atlanticum***

To estimate the active carbon transport by *P. atlanticum* through diel vertical migration we followed the methodology applied in Henschke *et al*. (2019) for this species. We have only considered the migratory population (52% of medium size colonies and 100% of large size colonies, see results section of the main text).

Wet weight (*WW*; g) of *P. atlanticum* colonies was estimated from the median colony length (*l*; mm) of the medium and large size colonies each year following the equation:

(1) $WW=0.0013l^{2}+0.0151l^{2}$

Respiration rate (*R*; ml O_2_/col.·h) was expressed by the allometric equation:

(2) $R=0.0046{WW}^{1.2284}$

Where *WW* corresponds to wet weight of the colony (g).

To calculate the respiratory carbon equivalent (*RC*; µg C/col.·d) from the respiration rate we used the equation from Al‐Mutairi and Landry (2001):

(3) $RC=R\cdot RQ\cdot\left( \frac{12}{22.4} \right)$

where R is the respiration rate (ml O_2_/col.·d), RQ is the respiratory quotient (1.16 for salps; see Mayzaud *et al.*, 2005), 12 is the molar weight of carbon (g/mol), and 22.4 is the molar volume (mol/L) of an ideal gas at standard pressure and temperature.

Following Henschke *et al.* (2019), dissolved organic carbon excretion was assumed to be approximately 31% of the carbon respired (RC). We applied a mortality estimation of 1%/d reported in areas of similar biomass (Henschke *et al*., 2019).

Daily faecal pellet production (*FP*; mg C/col.·d) was estimated by the equation:

(4) $FP=0.25\cdot CW$

where daily defecation rates are 25% of body carbon (Henschke *et al.*, 2019) and carbon weight (*CW*; mg C) was estimated to be 3.92% of wet weight of the colony for *P. atlanticum* (Lebrato and Jones, 2009).

Daily faecal pellet production allows to estimate the gut flux (*GF*; mg C/ col.·d; i.e. defecation or nondigested food), where 24 converts daily faecal pellet production to hourly;

(5) $GF=\frac{FP}{24}\cdot(GPT-DM)$

A gut passage time (GPT; h) of 1.43h for *P. atlanticum* (Perissinotto *et al.*, 2007) was considered. The time spent in downward migration (*DM*; h) was estimated using a mean swimming speed of 0.05 m/s (considering the speed range 0.03-0.07 m/s reported in Henschke *et al.* (2019)) and the distance between weighted mean depth during day and night (migration amplitude). DM was never higher than GPT.

Finally, we estimate the respiratory, excretory, mortality and gut flux contribution to the active carbon transport for *P. atlanctium* (µg C/m^3^·d) (Table S1). For the conversion of these values to mg C/m^2^·d the migration amplitude of each size class has been considered.

| **Year** | **Respiratory flux**  **(µg C/m^3^·d)** | **Excretory flux**  **(µg C/m^3^·d)** | **Mortality flux**  **(µg C/m^3^·d)** | **Gut flux**  **(µg C/m^3^·d)** | **Total carbon flux**  **(µg C/m^3^·d)** | **Total carbon flux**  **(mg C/m^2^·d)** |
| --- | --- | --- | --- | --- | --- | --- |
| **2017** | 0.66 | 0.21 | 3.45 | 0.93 | 5.26 | 0.56 |
| **2018** | 0.07 | 0.02 | 0.38 | 0.08 | 0.54 | 0.05 |

Table S1: Estimation of active downward carbon transport for *P. atlanticum* in the NW Mediterranean in 2017 and 2018.

The mean chl-*a* concentration in the upper 70 m of the water column (depth range that covers the weighted mean depth of all colony sizes during the night) was 0.65 ± 0.15 mg chl-*a*/ m^3^ in 2017 and 0.86 ± 0.56 mg chl-*a*/ m^3^ in 2018). Applying the C:Chl-*a* ratio of 47 (g:g) for the NW Mediterranean (Latasa *et al.*, 2005), the carbon transport by *P. atlanticum* through diel vertical migration represents a 0.01% and a 0.001% of the carbon concentration in the upper water layers in 2017 and in 2018, respectively.

REFERENCES

Al‐Mutairi, H. and Landry, M. R. (2001) Active export of carbon and nitrogen at station ALOHA by diel migrant zooplankton. *Deep Sea Res. Part II Top. Stud. Oceanogr.*, **48**, 2083–2103.

Henschke, N., Pakhomov, E. A., Kwong, L. E., Everett, J. D., Laiolo, L., Coghlan, A. R., and Suthers, I. M. (2019) Large vertical migrations of *Pyrosoma atlanticum* play an important role in active carbon transport. *J. Geophys. Res. Biogeosciences*, **124**, 1056–1070.

Latasa, M., Morán, X. A. G., Scharek, R., and Estrada, M. (2005) Estimating the carbon flux through main phytoplankton groups in the northwestern Mediterranean. *Limnol. Oceanogr.*, **50**, 1447–1458.

Lebrato, M. and Jones, D. O. B. (2009) Mass deposition event of *Pyrosoma atlanticum* carcasses off Ivory Coast (West Africa). *Limnol. Oceanogr.*, **54**, 1197–1209.

Mayzaud, P., Boutoute, M., Gasparini, S., Mousseau, L., and Lefevre, D. (2005) Respiration in marine zooplankton — the other side of the coin: CO2 production. *Limnol. Oceanogr.*, **50**, 291–298.

Perissinotto, R., Mayzaud, P., Nichols, P. D., and Labat, J. P. (2007) Grazing by *Pyrosoma atlanticum* (Tunicata, Thaliacea) in the south Indian Ocean. *Mar. Ecol. Prog. Ser.*, **330**, 1–11.
